# The Post-Anaesthesia *N*-acetylcysteine Cognitive Evaluation (PANACEA) trial: study protocol for a randomised controlled trial

**DOI:** 10.1186/s13063-016-1529-4

**Published:** 2016-08-09

**Authors:** David R. Skvarc, Olivia M. Dean, Linda K. Byrne, Laura J. Gray, Kathryn Ives, Stephen E. Lane, Matthew Lewis, Cameron Osborne, Richard Page, Douglas Stupart, Alyna Turner, Michael Berk, Andrew J. Marriott

**Affiliations:** 1 School of Psychology, Deakin University, 221 Burwood Highway, Burwood, 3215 VIC Australia; 2 Department of Anaesthesia, Perioperative Medicine and Pain Management, Barwon Health, Bellarine Street, Geelong, 3220 VIC Australia; 3 Innovations in Mental and Physical Health and Clinical Treatment (IMPACT) Strategic Research Centre, Deakin University/Barwon Health Clinical School, Pigdons Road, Waurn Ponds, 3216 VIC Australia; 4 School of Medicine, Deakin University, Pigdons Road, Waurn Ponds, 3216 VIC Australia; 5 Biostatistics Unit, Barwon Health, Bellarine Street, Geelong, 3220 VIC Australia; 6 Barwon Orthopaedic Research Unit, Bellarine Street, Geelong, 3220 VIC Australia; 7 Department of General Surgery, Barwon Health, Bellarine Street, Geelong, 3220 VIC Australia; 8 Aged Psychiatry Service, Caulfield Hospital, Alfred Health, 260 Kooyong Road, Caulfield, 3162 Victoria Australia

**Keywords:** Anaesthesia and cognitive deficit, Cognitive dysfunction, Anaesthetics, Surgery, Oxidative stress, *N*-acetylcysteine, Antioxidant, Surgical stress response, Delirium, Dementia

## Abstract

**Background:**

Some degree of cognitive decline after surgery occurs in as many as one quarter of elderly surgical patients, and this decline is associated with increased morbidity and mortality. Cognition may be affected across a range of domains, including memory, psychomotor skills, and executive function. Whilst the exact mechanisms of cognitive change after surgery are not precisely known, oxidative stress and subsequent neuroinflammation have been implicated. *N*-acetylcysteine (NAC) acts via multiple interrelated mechanisms to influence oxidative homeostasis, neuronal transmission, and inflammation. NAC has been shown to reduce oxidative stress and inflammation in both human and animal models. There is clinical evidence to suggest that NAC may be beneficial in preventing the cognitive decline associated with both acute physiological insults and dementia-related disorders. To date, no trials have examined perioperative NAC as a potential moderator of postoperative cognitive changes in the noncardiac surgery setting.

**Methods and design:**

This is a single-centre, randomised, double-blind, placebo-controlled clinical trial, with a between-group, repeated-measures, longitudinal design. The study will recruit 370 noncardiac surgical patients at the University Hospital Geelong, aged 60 years or older. Participants are randomly assigned to receive either NAC or placebo (1:1 ratio), and groups are stratified by age and surgery type. Participants undergo a series of neuropsychological tests prior to surgery, 7 days, 3 months, and 12 months post surgery. It is hypothesised that the perioperative administration of NAC will reduce the degree of postoperative cognitive changes at early and long-term follow-up, as measured by changes on individual measures of the neurocognitive battery, when compared with placebo. Serum samples are taken on the day of surgery and on day 2 post surgery to quantitate any changes in levels of biomarkers of inflammation and oxidative stress.

**Discussion:**

The PANACEA trial aims to examine the potential efficacy of perioperative NAC to reduce the severity of postoperative cognitive dysfunction in an elderly, noncardiac surgery population. This is an entirely novel approach to the prevention of postoperative cognitive dysfunction and will have high impact and translatable outcomes if NAC is found to be beneficial.

**Trial registration:**

The PANACEA trial has been registered with the Therapeutic Goods Administration, and the Australian New Zealand Clinical Trials Registry: ACTRN12614000411640; registered on 15 April 2014.

**Electronic supplementary material:**

The online version of this article (doi:10.1186/s13063-016-1529-4) contains supplementary material, which is available to authorized users.

## Background

Cognitive decline after surgery is common amongst elderly surgical patients [1–3]. Postoperative cognitive dysfunction (POCD) refers specifically to dysfunction in postoperative cognitive performance relative to presurgical performance; however, the exact threshold delineating when cognitive decline can be classified as POCD varies considerably within the literature [4]. One of the most robust studies places the early postoperative incidence rate of POCD at approximately 25 % of major surgery patients, aged over 60 years [1, 3, 4], and research suggests that patients aged at least 60 years are at significantly greater risk compared to younger patients [37]. Generally, cognitive decline after surgery manifests as subtle dysfunction across a number of cognitive domains, such as memory, attention, and psychomotor function. It may persist in some patients for months or years and herald the onset of a dementia process, to the significant detriment of quality of life [5–8]. Given the lack of consensus regarding the exact demarcation of POCD, it is useful to refer instead to an absolute difference in cognitive function after surgery controlling for baseline values, regardless of whether or not such a change in function meets the specifications for POCD.

At present, there are neither pharmacological interventions nor preventative agents for POCD. The role of oxidative stress and neuroinflammation as a result of a surgical stress response is well-characterised, and now provides a potentially modifiable target for therapeutic intervention [9–13]. Developing evidence suggests that there is a relationship between surgery and anaesthesia and the production of inflammatory signals and reactive oxygen species in the periphery and the brain which are associated with cognitive dysfunction [14–19].

### Why *N*-acetylcysteine?

*N*-acetylcysteine (NAC) provides the rate-limiting molecule, cysteine, for glutathione production. Glutathione is the major antioxidant species in the brain, enhancing the brain’s capacity to respond to oxidative stress conditions which are associated with cognitive deficits [20, 21]. NAC also mitigates the negative effects of inflammation on cognition, suppressing overactivation of the brain’s inflammatory cells and dysregulation of the key neurotransmitter glutamate [22–24]. Commonly used in cases of acetaminophen overdose to protect against liver damage [58], in recent decades NAC has been studied as an intervention agent for a wide variety of neuropsychiatric disorders including cocaine dependence, trichotillomania, schizophrenia, depression, and bipolar disorder [54, 59]. Importantly, NAC is also effective in reducing cognitive deficits in preclinical models of Alzheimer’s disease via modulation of inflammation and oxidative stress [25–28].

NAC has been found to be beneficial in the clinical setting for a variety of psychiatric, neurological, and medical conditions with cognitive sequelae, including late-stage Alzheimer’s disease [29–32]. However, the administration of NAC in these settings typically forms a part of a supplement cocktail containing other substances with putative cognitive modulation [29–32] and so an investigation of NAC in addition to regular treatment is necessary. Further, studies examining the efficacy of NAC as a cognitive modulator in a clinical population are typically heterogeneous concerning dose strength and regimen [59], thus making consensus difficult. NAC has demonstrated efficacy in reducing the elevations of markers of inflammation and oxidative stress seen after major surgery; markers which are now thought to be predictive of risk of cognitive sequelae [33–35]. No study to date has directly examined human cognition after perioperative administration of NAC. Hence, we propose NAC may protect against postsurgical cognitive decline in the at-risk population.

### Aims

#### Primary

- To investigate the effectiveness of NAC in reducing the severity of cognitive decline following major elective noncardiac surgery

#### Secondary

- To investigate the effectiveness of NAC in reducing biomarkers of inflammation and oxidative stress in the perioperative setting
- To examine correlations between changes in cognition and biomarkers
- To identify potential predictors of cognitive change following surgery

### Hypotheses

#### Primary

- That administration of NAC in the perioperative setting is superior to placebo for reducing the severity of early POCD, as measured by changes in performance on the psychomotor-attention composite of the CogState Brief Battery (CBB)

#### Secondary

- That administration of NAC in the perioperative setting is superior to placebo for reducing the severity of enduring cognitive change in the late postoperative period, as measured by changes in performance on the psychomotor-attention composite of the CBB 3 months post surgery, and 1 year post surgery
- That administration of NAC in the perioperative setting is superior to placebo for reducing the severity of cognitive change in the early postoperative period, as measured by changes on the Repeatable Battery for the Assessment of Neuropsychological Status (RBANS), the Controlled Oral Word Association Test (COWAT), and Trail Making Tests A and B (TMT) 7 days post surgery
- That administration of NAC in the perioperative setting is superior to placebo for reducing the severity of cognitive change in the late postoperative period, as measured by changes on the RBANS, COWAT, and TMT at 3 months post surgery, and 1 year post surgery
- That administration of NAC in the perioperative setting is superior to placebo for reducing serum levels of biomarkers of inflammation and oxidative stress in the postoperative phase
- That there is an inverse correlation between performance on the neuropsychological battery and serum biomarkers of oxidative stress, at baseline, in the postoperative period and at follow-up

## Methods and design

A single-centre, randomised, double-blind, placebo-controlled clinical trial. A total of 370 participants are randomised into either a placebo or treatment (NAC) group in a 1:1 ratio.

### Trial procedures

**Fig. 1:**
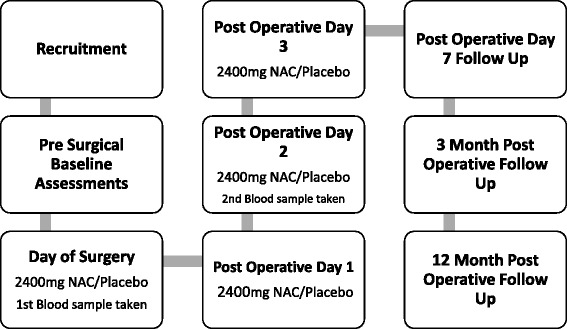
Trial flow chart. A graphical flow chart of the trial process, from recruitment and randomisation to the final data collection at 12 months

Participants undergo an initial recruitment and screening interview, a baseline assessment of cognition, mood, and quality of life prior to surgery, and follow-up visits at postsurgery day 2, day 7, 3 months, and 12 months. Serum sampling for biomarker assessment occurs immediately prior to surgery, and then again on postsurgery day 2 to correlate with peak oxidative stress levels (Fig. 1, Table 1). The SPIRIT checklist is provided as an Additional file 1.

**Table 1:** Timeline of instruments

Instruments	Recruitment	Prior to surgery	Day of surgery	POD1	POD2	POD3	POD7–14	3–4 months	12–13 months
MMSE^a^	X								
CBB^b^		X					X	X	X
RBANS^b^		X					X	X	X
COWAT^b^		X					X	X	X
TMT^b^		X					X	X	X
CAM^a^					X		X		
HADS^b^		X					X	X	X
WHOQOL-BREF^b^		X						X	X
NRS11^a^		X			X		X		
Modified Brief Pain Inventory^b^								X	
Neuropathic Pain Questionnaire^b^									X
Research blood sample – biomarkers^a^			X		X				

A table with assessment and data collection types listed as rows, and data collection points in columns. The corresponding collection point for each assessment is marked with an ‘X’”. This conforms with the SPIRIT guidelines (Additional file 1)

*CAM* Confusion Assessment Method, *CBB* CogState Brief Battery, *COWAT* Controlled Oral Word Association Test, *HADS* Hospital Anxiety and Depression Scale, *MMSE* Mini Mental State Exam, *NRS11* Numerical Rating Scale for Pain, *POD* postoperative day, *RBANS* Repeatable Battery for the Assessment of Neuropsychological Status, *TMT* Trail Making Tests A and B, *WHOQOL-BREF* World Health Organisation Quality of Life scale

^a^To be performed by trained research staff

^b^To be performed by research staff trained in the neuropsychological assessment

### Setting and participants

The study is being conducted at the University Hospital Geelong, a public teaching hospital that caters for the majority of surgical subspecialties other than neurosurgery, organ transplantation, and cosmetic plastic surgery. Patients undergoing major noncardiac surgical procedures of vascular; ear, nose, and throat; plastic; gynaecological; orthopaedic; urological; ophthalmological; and general surgery are invited to participate. Based upon data from the 2014 calendar year, there are over 1000 potentially eligible patients annually. Potential participants are identified and approached during presurgical outpatient clinics by experienced research nurses. Perspective participants are provided with an Information and Consent Form that must be signed and witnessed by a member of the research team before enrolment. Participant visits will take place at University Hospital or at the home of the participant where applicable.

### Regulatory approval

Barwon Health Human Research Ethics Committee and Deakin University Human Research Ethics Committee have provided ethics approval. Approval was required, and granted by, the Therapeutic Goods Administration (TGA) under the Clinical Trials Notification Scheme to conduct the study.

### Inclusion criteria

Patients aged 60 years or older who are undergoing major elective noncardiac surgery, defined as surgery expected to last at least 1 h in duration and requiring admission to hospital for at least two nights, are eligible for recruitment. This is in accordance with findings from the seminal International Study of POCD, where increased length of surgery was significantly associated with increased risk of POCD after approximately 1 h [1, 2].

### Exclusion criteria

Participants are ineligible to take part if they:

- Have known contraindications to NAC, including allergies and concomitant nitrate therapy
- Are unable to comply with the requirements of informed consent or the study protocol
- Are already taking NAC
- Are undergoing carotid endarterectomy, due to the high incidence of perioperative cerebrovascular accidents after this procedure [36]
- Are undergoing surgery for trauma
- Lack the capacity to consent to their own surgery

### Randomisation, allocation, and concealment

Once consent is confirmed, participants are randomised to a treatment group. Randomisation is stratified by surgery type (orthopaedic or nonorthopaedic) and age (less than 80 years or 80 years or older) to provide four strata for the study. The allocation code was produced by an independent researcher prior to the commencement of recruitment and was generated by a computer-generated list, using permuted block randomisation with variable block sizes. The allocation code is concealed to all trial personnel, with the exception of the dispensing pharmacist. The pharmacist allocates the participant number, based on the relevant stratum and holds the code if unblinding is required, but is not involved in data collection. All other relevant persons, including investigators, research assistants, research nurses, and participants are blind to the treatment allocation until the end of the data analysis.

To aid treatment concealment, all trial materials appear identical. Further, the placebos have been dusted with a nontherapeutic amount of NAC to assist with matching odours (given that NAC has a strong odour). The placebos and NAC capsules are identical in size, taste, and appearance. All trial medication is provided in identical bottles. Any unused medication is returned to the pharmacy for auditing.

Stratification by age enhances parity between groups in terms of its effects upon cognition, in accordance with established risk factors [5, 37]. Despite the relative safety and success of orthopaedic operations compared with nonorthopaedic operations, the risk of cognitive dysfunction is still significant [3]. Accordingly, orthopaedic patients represent an opportunity to examine potential cognitive change that is less likely attributable to other factors. Additionally, stratification by orthopaedic and nonorthopaedic counteracts the wide variability of reported POCD in the literature [38]. Meta-analyses suggest small cognitive improvements from pooled data yet individual studies note an incidence of POCD as high as 45 % [38]. It is widely held that this variability is due mostly to discrepancies in diagnostic criteria [39] but the concern remains that a significant subset of individuals within the orthopaedic cohort do experience cognitive improvement. The literature to date has not provided the means to identify them prospectively, but it has been suggested that a low cognitive baseline secondary to chronic inflammation, pain, opioid consumption, and restricted mobility, coupled with the potential for surgical remedy, is a potent confounder of postsurgical cognition [40]. This complex and not yet fully characterised surgical profile suggests that the broad classification of surgeries into orthopaedic and nonorthopaedic may help to minimise any confounding in the collected data, with negligible loss of power with respect to a nonstratified design.

### Dose and treatment regimen

Administration of NAC in a wide variety of medical settings has been shown to be safe [41, 42]. Whilst there is variation within the literature regarding dose and timing of NAC, recent reviews have suggested NAC administered per os at doses of 2400 mg/day to be efficacious in delivering demonstrable biomarker change with a negligible adverse effect profile [43, 44]. Kuyumcu et al. [33] showed improved oxidative status in abdominal surgery patients with a total dose of 2400 mg of NAC administered intravenously over 2 days prior to surgery. Dose regimens well in excess of 2400 mg/day have been safely explored with some regularity, but are likely to be burdensome during oral administration, a route which offers greater safety [62] and ease of administration than intravenous infusion. Bearing in mind the previously established effective doses, the expected duration of the surgical stress response [42], the oral bioavailability of NAC and the relative ease, safety and cost effectiveness of oral administration, we have chosen a regimen of 1200 mg NAC administered per os twice daily, consisting of two 600-mg capsules in the morning and again in the evening, starting on the day of surgery and continuing for 4 days to a total cumulative dose of 9600 mg. Administration takes place during the patients’ regular morning and evening drug rounds, facilitated by the ward staff and recorded from the patient chart by the research nurses. Placebo is administered in an identical fashion. Medication is dispensed by the pharmacy and administration coordinated by the research nurses, and recorded in the trial records for each participant. Unused medication is returned to the pharmacy for disposal. Episodes of nausea and vomiting are recorded by research staff.

### Assessments and information

Demographic data and details of participants’ medical history are recorded to explore potential risk factors and confounders, gathered from participant interview, chart review, and examination of medical history. The following variables are specifically sought to identify potential predictors of outcome: age, gender, participants who identify as having Aboriginal and Torres Strait Islander descent, educational attainment, chronic inflammatory diseases (such as rheumatoid arthritis), osteoarthritis and inflammatory bowel disease, medications with anti-inflammatory properties (including statins, NSAIDs and steroids), preexisting psychiatric conditions and/or medication, and vascular risk factors including diabetes, total cholesterol, smoking status, hypertension, atrial fibrillation, heart disease – ischaemic/cardiac failure, and previous transient ischaemic attack/stroke.

### Cognitive function measures

Cognitive function is assessed prior to surgery, 7 days post surgery, 3 months post surgery, and again 12 months post surgery. Cognitive dysfunction at approximately 1 week post surgery has been shown to be a significant predictor of further cognitive change in elderly postsurgical patients, and so comparisons of cognition at postoperative day (POD) 7 with preoperative baseline is considered the primary outcome [37]. A number of cognitive domains are measured using a selection of validated neuropsychological tests. All assessments are reliable and validated for the use in detecting cognitive change [45–51, 61, 62]. All assessments, with the exception of the CBB, are recorded in specially created Case Report Forms, and stored securely after the transcription of data into the secure computer database (Research Electronic Data Capture, REDCap). The CBB automatically uploads data into a secure database, and does not require paper records. All assessments are performed by trained research staff, supervised for compliance and quality by the senior clinicians. Psychomotor, attention, learning, and working memory domains are assessed with the computerised CBB. The CBB consists of four tasks: detection, identification, one-card learning, and one-card back. In the detection task, the participant is asked to press a button as soon as a displayed card is turned over. The psychomotor-attention composite is made up of the detection and identification tasks on the CBB and represents the reaction time of the participant, where higher scores indicate lower reaction speed and can be used to suggest the presence of impairment. The CBB has shown efficacy in detecting cognitive change in healthy participants over 12 months [60], and in clinical samples. [45]. Immediate memory, visuospatial, and delayed memory domains are assessed with the Repeatable Battery for the Assessment of Neuropsychological Status (RBANS) [46]. The RBANS consists of 12 subtests measuring immediate and delayed verbal and visuospatial memory, and is commonly used for the detection of cognitive change [46]. Alternate forms are used at each assessment.

Verbal fluency and executive function is assessed with the Controlled Oral Word Association Test (COWAT). Over three attempts, participants are given 1 minute to list as many words beginning with a specified letter. Errors and perseverations are recorded [47].

Visuospatial and executive function is assessed with the Trail Making Tests A and B (TMT). Participants are asked to connect a series of numbered scattered dots in sequential order. Only numbered dots are used in Test A; alternating numbers and letters are used in Test B [48].

### Delirium assessment

Postoperative delirium typically manifests between 24 and 72 h post surgery, and may last up to several days [61]. Delirium is assessed on POD 2 and POD 7 using the short form of the Confusion Assessment Method instrument (CAM) [49].

### Mood disorder measurement

‘Depression and Anxiety’ is assessed prior to surgery, 7 days post surgery, 3 months post surgery, and 12 months post surgery using the HADS questionnaire. The HADS is a brief self-report Likert scale where participants report how well a depressive or anxious symptoms describes them in the past 7 days (e.g. ‘I felt tense or “wound up”’ [50]).

### Quality of life measurement

Quality of life is assessed prior to the operation, at 3 months post surgery, and again at 12 months post surgery using the World Health Organisation Quality of Life – BREF questionnaire (WHOQOL-BREF) [51].

### Blood levels of inflammation and oxidative stress

Serum samples are collected during placement of an intravenous line during surgery (baseline), and again in conjunction with routine postoperative bloods on postsurgery day 2. This is timed to capture the approximate time of peak postoperative inflammatory markers of approximately 24–72 h post surgery [52]. Blood samples (30 ml) are taken by research staff and spun in equal portions into separate plasma, serum, and whole blood aliquots. These are then stored onsite at −80 °C in preparation for analysis by research staff at the end of data collection.

Whilst surgery and anaesthesia are associated with a systemic inflammatory response, patients who develop POCD tend to show higher levels of inflammatory markers, including interleukin-(IL)1β [52, 53], relative to those with intact cognition. Similarly, markers of oxidative stress, previously correlated to neuroinflammation, may be upregulated in POCD [52]. Circulating inflammatory factors are thought to potentiate neuroinflammation through stimulation of microglia, which produce both pro-inflammatory cytokines and potent reactive oxygen species and are instrumental in neuronal dysfunction [45]. Thus, measurement of peripheral inflammation and oxidative damage are reflective of not only systemic inflammation in the postoperative context but also central neuroinflammatory processes. We intend to examine a panel of related biological correlates, focusing on inflammatory cytokines, the primary CNS antioxidant species and markers of oxidative damage to lipid, DNA, and protein. Unspecified consent has been requested to allow for the exploration of relevant biological markers should they be identified during the course of the trial. Changes in peripheral markers will be correlated to clinical improvements.

### Perioperative assessment of pain

A numerical rating of perioperative pain experienced by the participants is recorded at baseline, POD 2 and POD 7 using the Numerical Rating Scale-11. Additionally, the Modified Brief Pain Inventory is conducted at 3 months, and the Neuropathic Pain Questionnaire is conducted at 12 months.

### Outcome variables

Primary outcome variable:

- A between-group comparison of cognitive performance in the early postoperative period (day 7), as measured using the psychomotor-attention composite score of the CogState Brief Battery (CBB)

Secondary outcomes:

- Between-group comparison of cognitive performance in the early and late postoperative period, as measured using the extended neuropsychological battery, at day 7, 3 months, and 1 year post surgery. Comparisons at each time point will be made to scores obtained at presurgical baseline
- Between-group comparison of mood disorder, as measured using the Hospital Anxiety and Depression Scale (HADS), at day 7, 3 months, and 1 year post surgery. Comparisons at each time point will be made to raw scores obtained at presurgical baseline
- Between-group comparison of quality of life, as measured using the World Health Organisation Quality of Life – BREF (WHOQOL-BREF) scale, at 3 months, and 1 year post surgery. Comparisons at each time point will be made to raw scores obtained at presurgical baseline
- Between-group comparison of delirium, as measured using the Confusion Assessment Method (CAM), on postsurgery days 2 and 7. Incidents of delirium are recorded dichotomously
- Between-group comparison of postoperative pain, as measured using the Numerical Rating Scale (NRS) on postsurgery days 2 and 7; the Modified Brief Pain Inventory at 3 months, and the Neuropathic Pain Questionnaire at 12 months post surgery
- Between-group comparison of levels of serum neuroinflammation and oxidative stress, including but not restricted to, tumour necrosis factor (TNF)α, IL-1β IL-6, S100β, malondialdehyde, and reduced and oxidised glutathione on postsurgery day 2

### Withdrawal

Participation is entirely voluntary and consent may be withdrawn at any stage. Withdrawal reasons are documented and recorded. Participants are not considered to have withdrawn if they are lost to follow-up, and research staff will attempt to reconnect with participants for subsequent assessments. For example, a participant who is unable to be contacted for the 3-month follow-up is recorded as a missed session, but research staff will attempt to contact the participant for the 12-month follow-up. Participants are withdrawn from treatment and/or assessment if they:

- Withdraw consent for participation
- Are unblinded for any purpose prior to the end of the treatment period
- Are requested to withdraw by their treating medical team due to interference with their appropriate clinical management
- Require subsequent surgery or anaesthesia prior to measurement of the primary outcome variable

### Adverse events

Adverse events (AEs), serious adverse events (SAEs), and suspected unexpected serious adverse reaction (SUSAR) monitoring and reporting are conducted in strict accordance with the processes established by both governing ethics committees.

AEs are recorded from the time that informed consent has been obtained until the end of the 12-month study period. For each, the following parameters are described:

- The investigator’s assessment of the causal relationship between the event and the investigational treatment
- A description of the AE, including its nature, start and stop date, action taken with regards to the investigational treatment, intensity and outcome
- A statement if the AE fulfils the criteria for an SAE or a SUSAR

Intensity is determined according to the following categories:

- Mild: an AE which is easily tolerated by the participant, causing minimal discomfort and not interfering with everyday activities
- Moderate: an AE which is sufficiently discomforting to interfere with everyday activities
- Severe: an AE which is incapacitating and prevents everyday activities and/or requires therapeutic intervention, i.e. use of a prescription drug or hospitalisation

### Follow-up of adverse events and SUSARs

AEs are followed up until either they are resolved, or persist despite cessation of the trial medication and its elimination from the body (given that the half-life of NAC is roughly 6 h, this is estimated to take 2 days). AEs may be followed up for longer at the discretion of the investigators. 

### Reporting of serious adverse events and SUSARs

Reporting of SAEs to regulatory authorities is done within the requisite 24-hour period by an investigator in accordance with local regulations. A copy of the SAE report is sent to the Barwon Health Human Research and Ethics Committee, the sponsor and the principal investigator. If unblinding is required, a report will also be sent to the Therapeutic Goods Administration.

### Mortality reporting

Regardless of causality or which randomisation arm the participant is assigned to, all cases of mortality are reported to the Data Safety Management Board and regulatory authorities.

### Termination and safety

It is intended that the trial will run for the projected duration of 4 years from recruitment of the first patient. A DSMB has been established for this study and is independent of the investigators. Investigators provide intermittent reports to the DSMB with any information relating to safety analyses, and monitor instances of adverse. The DSMB provides recommendations regarding the continuation of the study.

### Sample size determination

Due to variations in definitions and cut-off criteria, measuring the effects of POCD, in the context of previous literature, is extremely difficult. Additionally, there is no literature available examining the use of the CBB for the detection of POCD, despite being theoretically suited to the task. However, literature that examines differences between healthy controls and mild cognitive impairment (MCI) provides a close approximation of the quantifiable effects of cognitive change after surgery. An examination of the available research utilising the CBB provides an estimated effect size of cognitive change in comparison to healthy controls, MCI, and Alzheimer’s disease participants.

The literature suggests a strong correlation between the two tasks of the psychomotor-attention composite, detection and identification, for simplicity’s sake assumed to be in the region of *r* = 0.8 is expected (for example, see the similarities present on scores from the research of Hammers et al., 2012; [55] and Lim et al., 2012 [56]). Further, the variances for pre and postsurgery will be standardised to 1. The variance of the composite is calculated as the sum of pre and postsurgical variances plus twice the correlation of the composite tasks; 2 + 2 (0.8) = 3.6. Therefore, to estimate the effect of MCI (and by extension, cognitive change after surgery), we divide the combined effect size for the psychomotor-attention composite by the standard deviation of the effect size, that is the square root of the variance of the composite. From Lim et al., the effect of MCI size of the change in reaction speed for the detection and identification tasks were −0.33 and −0.63, respectively, giving a total reduction in reaction speed effect size of −0.96. Therefore, the total effect of MCI (and by extension, cognitive change after surgery) upon the psychomotor-attention composite is equal to: −0.96/√3.6 = −0.51. Given that NAC is unlikely to improve cognition beyond baseline estimates, it is proposed that NAC may instead ameliorate the severity of cognitive dysfunction compared with placebo. Given the limited available data, we have attempted to estimate an approximate protective effect based on the use of NAC in analogous conditions, such as traumatic brain injury [63]. Additionally, it is unrealistic to assume complete prevention of cognitive dysfunction. Therefore, a relative reduction in severity of cognitive dysfunction of 50 % represents an appropriate compromise to calculate the power. Given the likelihood of significant correlation in pre and postsurgery cognition scores, and in the absence of any identifiable correlation size, it is prudent to include an estimation of correlation patterns in the medium range (*r* = 0.50). A protective effect of NAC of 50 % would then set the expected effect of postoperative cognitive change as modulated by NAC at = −0.255. Assuming an alpha level of 0.05, power of 80 %, and a pre and postsurgical score correlation of 0.5, a sample size of 370 participants will have 80 % power to detect a minimum absolute effect size of 0.255 using an analysis of covariance (ANCOVA) model. This sample size includes approximately equal representation across stratification by age and surgery type for intervention.

### Primary analysis

The primary study hypothesis states that the perioperative administration of NAC is superior to placebo in reducing the severity of postoperative dysfunction, as measured by the CBB at POD 7, and compared with baseline. Analysis of the primary outcome will be via ANCOVA, utilising the baseline psychomotor-attention composite score as a covariate. Assumptions of the ANCOVA will be examined through standard tests and residual plots, with transformation of variables being considered if normality of the residuals is not met. The psychomotor-attention composite is made up of the detection and identification tasks on the CBB and represents the reaction time of the participant, where higher scores indicate lower reaction speed and can be used to suggest the presence of impairment. The composite score is calculated by standardising each task score and adding them together. All analyses will be performed as intent-to-treat, in order to best emulate practice settings.

### Secondary analyses

Linear mixed-model ANCOVA will be conducted to model the differences in cognitive changes between treatment groups by fitting a linear curve to represent the relationship between performance on tasks and time point. The magnitude of the differences between treatment groups will be expressed as Cohen’s *d*.

Between-group differences on the individual tasks of the neuropsychological battery will be examined. Similar to the primary analysis, an ANCOVA will be used to examine the performance of participants on each of the neuropsychological battery tasks. Included in the analyses will be the individual CBB tasks, detection, identification, one-card back, and one-card learning; RBANS tasks, the COWAT, and Trail Making Tests A and B. Data will be represented as a linear mixed-effects model, comparing the performance of the NAC group compared to the control group over time. The magnitude of difference between treatment groups will be calculated as Cohen’s *d*.

Between-group differences on measures of delirium and mood dysfunction will be analysed using a chi-square test using the CAM and HADS measures. Participants will be classified dichotomously as displaying symptoms of delirium or mood dysfunction according to established cut-off points; 11 points on either anxiety or depression for the HADS, or fulfilment of delirium criteria according to the CAM.

Between-group differences on measures of quality of life will be analysed using an ANCOVA to examine changes over the course of the trial, controlling for baseline values as a covariate. Between-group differences will be examined for change across time from baseline scores to 3 months and 12 months. A linear mixed-effects model will be used to represent the data comparing treatment groups, with the magnitude between treatment groups expressed as Cohen’s *d*.

Between-group differences in serum levels of biological markers will be examined prior to surgery and at POD2 in order to capture the surgical stress response. An ANCOVA will be performed to examine the between-group differences over time, with baseline values acting as a covariate. Data will be graphed using a linear mixed-effects model, with the magnitude of difference between treatments groups expressed as Cohen’s *d*.

A bivariate correlation analysis exploring serum biological marker levels and cognitive function will be calculated for each of the battery tasks.

### Exploratory analyses

Subgroup analyses will be undertaken to examine the role of variables that may contribute to risk factors associated with POCD, such as demographic information, health and medical history, socioeconomic status, and other data collected during the study. These subgroup analyses can be examined using a multilevel modelling approach. For example, by treating the presence of POCD in a participant as an outcome level, it will be possible to examine the relative contributions of each independent variable to POCD (e.g. varying treatment effects for differing levels of demographics). This approach can also be expanded to further examine other participant outcomes, such as the presence of delirium, mood dysfunction, or a significant change in quality of life. For exploratory analyses focussed upon proportionate data, such as comparisons of dichotomous delirium or mood dysfunction outcomes, McNemars’ test can be used to examine within-groups differences across time points. The role of perioperative pain upon POCD may also be explored as a potential cognitive trajectory modulator.

### Missing or incomplete data

All participants recruited into the study, regardless of treatment group, are to have as much data as possible recorded, as fully as possible in accordance with this protocol. Missing data is monitored so as to avoid bias by attrition or patterns of missing data. All cases of missing data, including instances of nonretention of participants or withdrawal of consent, are to be recorded by research staff. Where data is missing, the frequency and reasons for missing data are to be recorded. Missing data is to be examined to see if there are patterns informative to the study; for example, if certain variables are missed in a systematic way by an identifiable pattern of participant.

In situations where data is missing, for example with participants who are lost to the 12-month follow-up, the data collected up to that point should still be used provided the participant has not withdrawn their consent or requested that the information be removed. If missing data is considered Missing at Random (MAR, i.e. without a pattern), then multiple imputation methods should be used to estimate missing values. The accepted minimum number of imputations is five, to estimate the missing data from existing data. This can be achieved using statistical software. If data appears to be missing in a way that is not random (Missing Not At Random, MNAR), then this data can be examined for potential biases or patterns statistically, and missing values can then be estimated using monotonistic methods.

## Discussion

Our study is the first randomised controlled clinical trial on the efficacy of perioperative NAC administration upon cognition. Because the trial will aim to recruit 370 participants and primarily examine the efficacy and safety of NAC, we consider this to be a phase 2 trial.

Australia’s population is ageing, and will continue to require greater numbers of surgical procedures. The commonness of cognitive dysfunction after surgery [1, 3, 4, 38], and the significantly increased risk of expedited morbidity and mortality [5–8], provide a strong community motivation to investigate potential interventions and it is hoped that NAC may fulfil that need.

The PANACEA trial is not without limitations, particularly the risk of attrition common in longitudinal studies. The potential for increased risks of nausea and vomiting may also result in participant withdrawal. Whilst all reasonable attempts to reduce the influence of attrition bias are employed, the possibility cannot be ruled out.

### Trial status

The PANACEA trial commenced recruitment in March 2015. The study has been registered with the Therapeutic Goods Administration, and the Australian New Zealand Clinical Trials Register (ANZCTR): ACTRN12614000411640. The study is performed in accordance with ethical and good clinical practice principles detailed by the Declaration of Helsinki and International Conference on Harmonisation [57].

## Abbreviations

AE, adverse event; ANCOVA, analysis of covariance; CAM, Confusion Assessment Method; CBB, CogState Brief Battery; COWAT, Controlled Oral Word Association Test; DSMB, Data Safety Monitoring Board; HADS, Hospital Anxiety and Depression Scale; IL, interleukin; MCI, mild cognitive impairment; NAC, *N*-acetylcysteine; NSAIDs, nonsteroidal anti-inflammatory drugs; PANACEA, Post-Anaesthesia *N*-acetylcysteine Cognitive Evaluation; POCD, postoperative cognitive dysfunction; POD, postoperative day; RBANS, Repeatable Battery for the Assessment of Neuropsychological Status; SAE, serious adverse event; TMT, Trail Making Test; WHOQOL-BREF, World Health Organisation Quality of Life-BREF

## Additional file

Additional file 1: SPIRIT Checklist – PANACEA. (PDF 106 kb)
